# Supplementary material for: Mutational Disruption of TP53: A Structural Approach to Understanding Chemoresistance
Source: Int J Mol Sci. 2025 Sep 18;26(18):9135. doi: 10.3390/ijms26189135 (PMC12470219; doi:10.3390/ijms26189135)
Supplement: Supplementary file 1 [file ijms-26-09135-s001.zip › supplentary_fig.pdf]

Mutational Disruption of TP53: A Structural Approach to Understanding Chemoresistance

Ali F. Alsulami

Department of Biochemistry, Faculty of Science, King Abdulaziz University, Jeddah 21589, Saudi Arabia; afmalsulami1@kau.edu.sa

Supplementary Materials

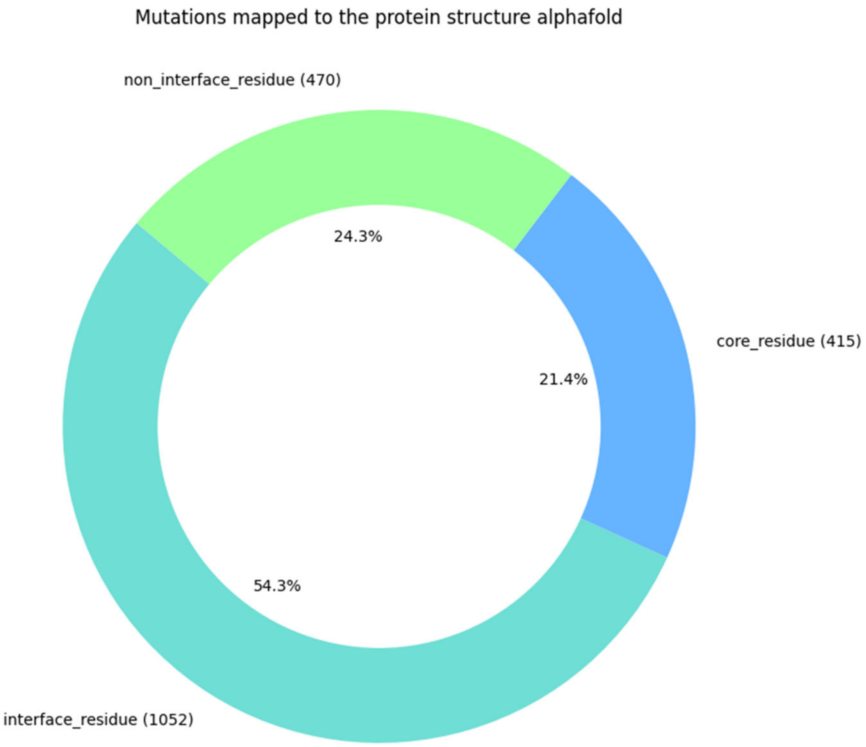

Figure S1. Represents the mapping of mutations on the AlphaFold predicted model

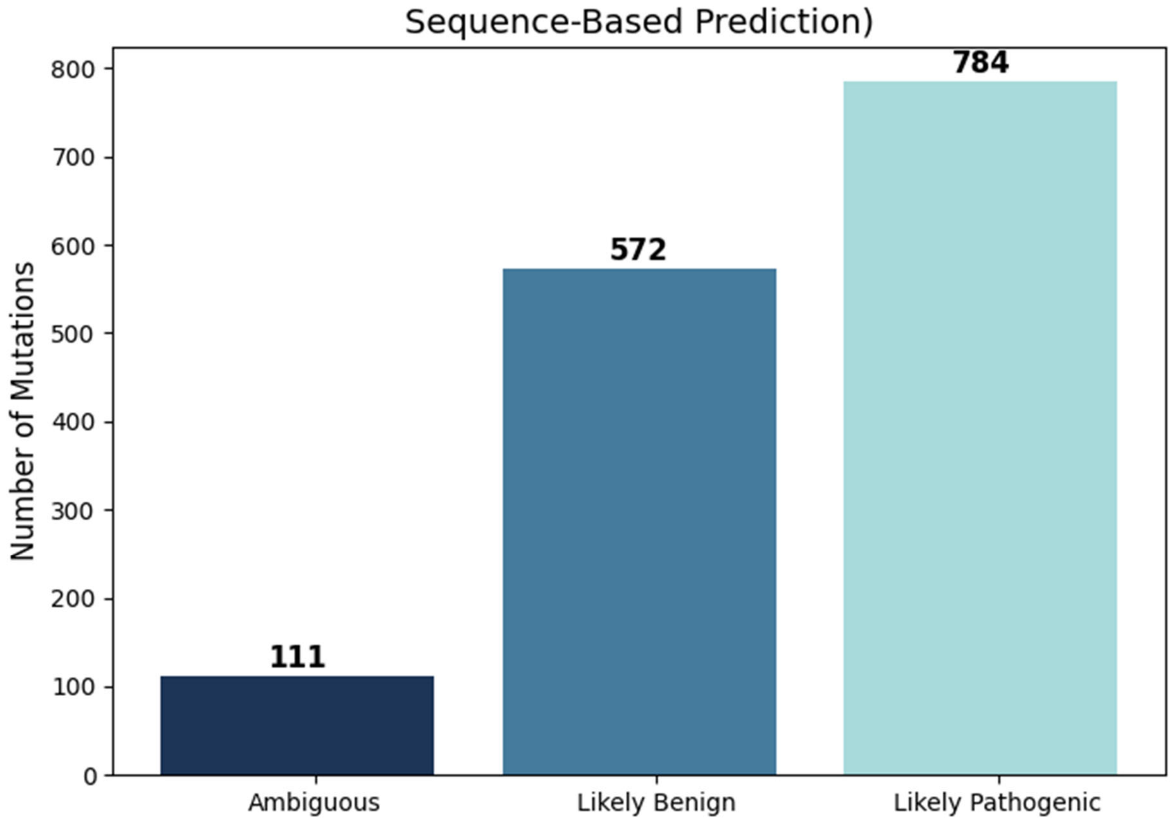

Figure S2. Classification of TP53 mutations reported in COSMIC into pathogenic and benign categories using AlphaMissense.
